# Genome-Wide Analysis of *NLR* Disease Resistance Genes in an Updated Reference Genome of Barley

**DOI:** 10.3389/fgene.2021.694682

**Published:** 2021-05-24

**Authors:** Qian Li, Xing-Mei Jiang, Zhu-Qing Shao

**Affiliations:** School of Life Sciences, Nanjing University, Nanjing, China

**Keywords:** barley, *NLR* gene, disease resistance, gene family, evolutionary analysis

## Abstract

Barley is one of the top 10 crop plants in the world. During its whole lifespan, barley is frequently infected by various pathogens. In this study, we performed genome-wide analysis of the largest group of plant disease resistance (*R*) genes, the nucleotide binding site–leucine-rich repeat receptor (*NLR*) gene, in an updated barley genome. A total of 468 *NLR* genes were identified from the improved barley genome, including one RNL subclass and 467 CNL subclass genes. Proteins of 43 barley *CNL* genes were shown to contain 25 different integrated domains, including WRKY and BED. The *NLR* gene number identified in this study is much larger than previously reported results in earlier versions of barley genomes, and only slightly fewer than that in the diploid wheat *Triticum urartu*. Barley Chromosome 7 contains the largest number of 112 *NLR* genes, which equals to seven times of the number of *NLR* genes on Chromosome 4. The majority of *NLR* genes (68%) are located in multigene clusters. Phylogenetic analysis revealed that at least 18 ancestral *CNL* lineages were presented in the common ancestor of barley, *T. urartu* and *Arabidopsis thaliana*. Among them fifteen lineages expanded to 533 sub-lineages prior to the divergence of barley and *T. urartu*. The barley genome inherited 356 of these sub-lineages and duplicated to the 467 *CNL* genes detected in this study. Overall, our study provides an updated profile of barley *NLR* genes, which should serve as a fundamental resource for functional gene mining and molecular breeding of barley.

## Introduction

Plants are consistently challenged by various pathogens during its whole lifespan. A two-layered immune system has been developed along the plant long-term evolution to defense infectious pathogens from environments (Wang et al., 2020; Zhang J. et al., 2020). The first layer immune system can recognize pathogen-associated molecular patterns (PAMPs) through plant cell surface-localized receptors, which induce PAMP-triggered immunity (PTI) (Wang et al., 2020; Zhang J. et al., 2020). Some pathogens can release effector proteins into plant cells to dampen signal transduction of PTI (Wang et al., 2020; Zhang J. et al., 2020). In response, the second layer immune system is required to detect those effectors, through proteins encoded by intracellular disease resistance genes (*R* genes), which induce effector-triggered immunity (ETI) (Wang et al., 2020; Zhang J. et al., 2020). Several types of *R* genes have been identified in the past twenty years. Among them, the nucleotide binding site (NBS)-leucine-rich repeat (LRR) receptor (*NBS-LRR*, also termed as *NLR*) gene family comprise the majority of *R* genes identified to date (Kourelis and van der Hoorn, 2018).

*NLR* genes are specifically discovered in the plant lineage, and their origin could be traced back to the common ancestor of all green plants (Shao et al., 2019). Phylogenetic analysis suggested that *NLR* genes had diverged into different subclasses prior to the divergence of green plants (Shao et al., 2019). Distinct N-terminal protein domains, including Toll/Interleukin-1 receptor (TIR) domain, Coiled-coil (CC) domain and Resistance to powdery mildew8 (RPW8) domain, have been found from different *NLR* subclasses. Accordingly, the three *NLR* subclasses were named as *TIR-NLR* (*TNL*), *CC-NLR* (*CNL*), and *RPW8-NLR* (*RNL*), respectively (Meyers et al., 2003; Shao et al., 2016). Genome-wide analysis revealed that angiosperm genomes contain abundant and variable number of *NLR* genes. For example, the *NLR* gene number in Poaceae species ranges from 145 in *Zea mays* to 2298 in *Triticum aestivum* (Liu et al., 2021). *NLR* subclasses composition is also different among angiosperm species. Generally, all monocots and most sequenced magnoliids lack the *TNL* subclasses, whereas the majority of dicot species genomes have all three *NLR* subclasses (Liu et al., 2021).

Defining the *NLR* gene composition in a species is not only helpful for exploring the evolutionary pattern of *NLR* gene family, but also important for mining and utilization of functional *NLR* genes. Genome-wide *NLR* gene analysis have greatly promoted functional *NLR* gene cloning in several crops. For example, dozens of *NLR* genes against rice blast have been identified from rice and other Poaceae species by genome-wide identification and comparative genomic analysis (Yang et al., 2013; Wang et al., 2019). Recently, analysis of multiple wheat genomes contributed to the successful cloning of *Sm1*, a *R* gene resistant to the orange wheat blossom midge (OWBM, *Sitodiplosis mosellana* Géhin) (Walkowiak et al., 2020).

Cultivated barley, *Hordeum vulgar*e L. ssp. *vulgare*, is one of the top ten crop plants in the world^1^. The product is not only used for animal feeding and malt production, but also serves as a major food staple in many contraries and regions of the world. In 2018, the world-wide production of barley ranks the fourth among all cereal crops (FAOSTAT, 2018). However, like other cereals, barley is also frequently infected by a variety of pathogens. Dozens of different diseases caused by fungi, bacteria, viruses and nematodes have been reported in barley, which result in significant yield reduction and poor grain quality (Murray and Brennan, 2009). However, only a few *R* genes have been identified from barley, including the *Rph1*, *Rph15* and some *MLA* alleles (Seeholzer et al., 2010; Chen et al., 2021).

Two previous studies performed genome-wide analyses of *NLR* genes, using earlier versions of the barley genome assemblies by short-read sequence strategy, and only 100 or so *NLR* genes were identified (Andersen et al., 2016; Habachi-Houimli et al., 2018). These numbers are much smaller than those in diploid wheat genomes, which have more than 500 *NLR* genes (Liu et al., 2021). A recent study reported a newly assembly of barley genome of the cultivar Morex by long-read sequencing technology (Mascher et al., 2021). Investigation of the gene composition in the *MLA* locus revealed that three tandem *CNL* genes at this locus were missed in the earlier assemblies but present in the new assembly (Mascher et al., 2021). The quality of genome assembly and annotation is critical to genome-wide analysis to gene families, especially to *R* genes with resembled repeats and duplicates. The above comparison indicated that the *NLR* genes in barley genome might be greatly underestimated. In this study, we performed a genome-wide *NLR* gene analysis based on the newly released barley genome, which should provide full and more comprehensive information of *NLR* genes in this important crop.

## Materials and Methods

### Data Used in This Study

The protein coding DNA sequences, amino acid sequences and gff3 annotation files of the reference genome sequence assembly of barley cv. Morex V3. were downloaded from the electronic data archive library (e!DAL)^2^ (Mascher et al., 2021). The *Arabidopsis thaliana NLR* genes were retrieved from our previous study (Zhang et al., 2016). The *T. urartu NLR* genes were downloaded from the angiosperm *NLR* atlas ANNA^3^ (Liu et al., 2021).

### Identification and Classification of Barley *NLR* Genes

*NLR* gene identification in the barley genome was performed using BLAST and hidden Markov models search (HMMsearch) methods as described previously (Shao et al., 2014). Briefly, the amino acid sequence of the NBS (also named as NB-ARC) domain was downloaded from the Pfam database (accession number: PF00931) and used as a query to search for NLR proteins using the BLASTp program of the NCBI BLAST software, with expectation value (*E*-value) setting to 1.0. Simultaneously, the HMM profile of the NBS domain was used as a query to perform a HMMsearch against protein sequences of barley with an *E*-value setting of 1.0. Then, the results from the two methods were merged together. A round of HMMscan was performed for all the obtained hits against the Pfam-A database (*E*-value set to 0.0001) to confirm the presence of the NBS domain. Genes do not encode a conserved NBS domain were removed from the datasets. The non-redundant candidate sequences were subjected to the online NCBI Conserved Domains Database (CDD) to identify the CC, RPW8, LRR and other integrated domains. MEME analysis (Bailey et al., 2009) was performed to discover conserved motifs in the NBS domain of the identified *NLR* genes. The number of displayed motifs was set to 20 with all other parameters default settings as described by Nepal and Benson (2015).

### Chromosomal Distribution of Barley *NLR* Genes

Chromosomal distribution of barley *NLR* genes was analyzed as described previously (Ameline-Torregrosa et al., 2008). The barley gff3 annotation file was parsed to extract the genomic locations of identified *NLR* genes. A sliding window analysis was performed with a window size of 250 kb. If two successive annotated *NLR* genes were located within 250 kb on a chromosome, they were considered as clustered.

### Phylogenetic Analysis

Sequence alignment and phylogenetic analysis were performed as described by Shao et al. (2014); Zhang Y. M. et al. (2020). Briefly, amino acid sequences of the conserved NBS domain encoded by barley *NLR* genes were retrieved and aligned using ClustalW with default options, and then manually corrected in MEGA 7.0 (Kumar et al., 2016). Too short or extremely divergent sequences were excluded from the analysis. Phylogenetic analysis was carried out by IQ-TREE using the maximum likelihood method (Nguyen et al., 2015) after selecting the best-fit model by ModelFinder (Kalyaanamoorthy et al., 2017). Branch support values were estimated using SH-aLRT and UFBoot2 tests (Minh et al., 2013). The phylogeny was reconciled as previously described (Shao et al., 2014) to reconstruction the ancestral state of the *NLR* genes.

### Synteny and Gene Duplication Analysis

Pair-wise all-against-all BLAST was performed for the barley protein sequences. The obtained results and the gff3 annotation file were then subjected to MCScanX for determination of the gene duplication type (Wang et al., 2012). Microsynteny relationships were analyzed and displayed using Tbtools (Chen et al., 2020).

## Results

### Barley Genome Contains Over 400 *NLR* Genes

By surveying the annotated protein coding genes of the improved barley genome, a total of 468 *NLR* genes were identified (Supplementary Table 1), accounting for approximately 0.7% of the more than 62,648 annotated protein coding genes. The number of *NLR* genes identified from the improved barley genome is three to fourfold larger than those reported in the previous studies (Andersen et al., 2016; Habachi-Houimli et al., 2018). To assign the identified *NLR* genes into different subclasses, a BLASTp analysis was performed for all obtained *NLR* genes against the well-defined *A. thaliana NLR* proteins (Zhang et al., 2016). The results showed that the 468 barley *NLR* genes comprise one *RNL* and 467 *CNL* genes. *TNL* genes were not detected in the barley genome, which is consistent with the notion that *TNL* genes are lost in the common ancestor of monocots (Collier et al., 2011; Shao et al., 2016; Liu et al., 2021).

**FIGURE 1:**
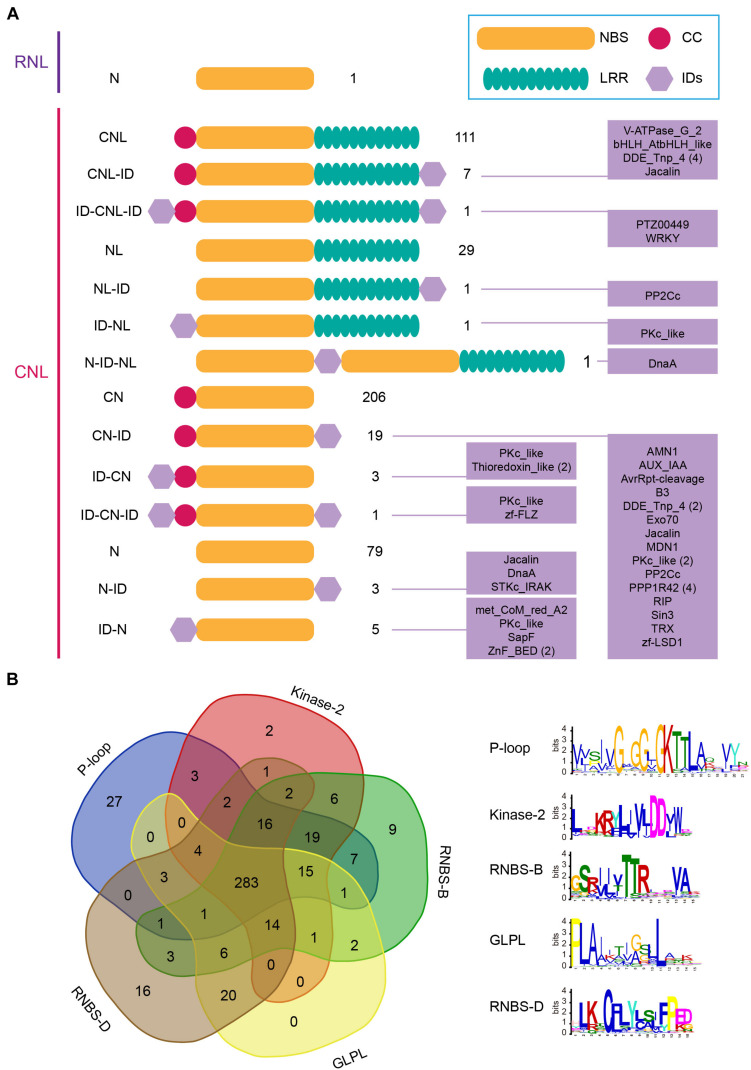
Identification and classification of barley *NLR* genes. **(A)** Domain compositions and arrangements of proteins encoded by 468 barley *NLR* genes. **(B)** Presence of five key motifs in the amino acid sequence of the NBS domain of 468 barley *NLR* genes.

Domain structure analysis revealed high structure diversity of barley NLR proteins. Proteins encoded by the 467 *CNL* genes could be classified into 14 groups according to their domain composition and arrangement (Figure 1A). Among them, only 119 *CNL* genes encode intact CNL proteins that contain both the N-terminal CC domain and the C-terminal LRR domain, in addition to the central NBS domain (Figure 1A). Seven of these intact *CNL* genes encode additional integrated domains (IDs) at the C-terminal, forming a CNL-ID structure; and one has both N-terminal and C-terminal IDs, forming a ID-CNL-ID structure (Figure 1A). There are 32 *CNL* genes encoding proteins without the N-terminal CC domain. The presence/absence of additional IDs at N-terminal and/or C-terminal further separated these genes into NL (29 genes), NL-ID (1), ID-NL (1), and N-ID-NL (1) groups (Figure 1A). There are 229 *CNL* genes encoding proteins without the C-terminal LRR domain, including 206 CN, 19 CN-ID, three ID-CN and one ID-CN-ID protein (Figure 1A). 87 *CNL* genes lost both the N-terminal CC domain and the C-terminal LRR domain (Figure 1A). Five and three of them fused IDs at N-terminal and C-terminal, respectively (Figure 1A). A total of 25 different IDs were detected from 43 barley NLR proteins, accounting for 9% of all NLR proteins. Several of them have been shown to have important function in NLR protein function, e.g., WRKY and BED family domains.

We detected the presence of five key motifs in the amino acid sequence of NBS domain by MEME analysis (Bailey et al., 2009). The result showed that the five motifs P-loop, Kinase- 2, RNBS-B, GLPL, and RNBS-D are readily detected and highly conserved in barley *NLR* proteins as reported in other angiosperms (Shao et al., 2016). Likewise, frequent losses of motifs were detected in many barley *NLR* proteins. Among the 468 NLR proteins, only 283 preserve all five motifs, accounting for 60% of all NLR proteins (Figure 1B). In contrast, nearly 40% NLR proteins lost at least one key motif in the NBS domain (Figure 1B).

### A Majority of Barley *NLR* Genes Are Presented in Cluster on Chromosomes

**FIGURE 2:**
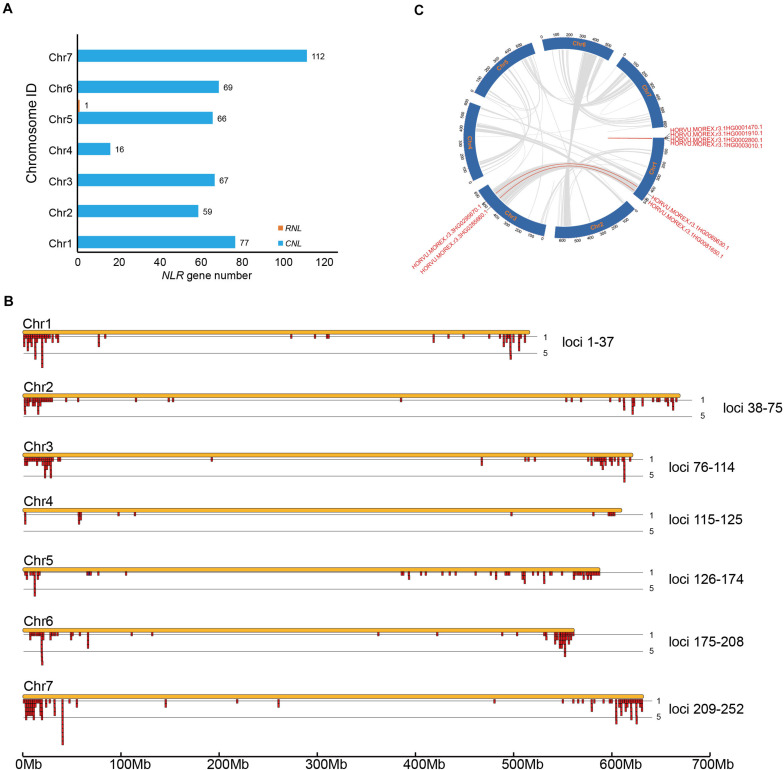
Chromosomal distribution of barley *NLR* genes. **(A)** NLR gene number variation among barley genomes. **(B)** Physical locations of *NLR* genes on barley chromosomes. *NLR* genes within an interval less than 250 kb were treated as a cluster (Ameline-Torregrosa et al., 2008). **(C)** Syntenic relationship of the eight segmental-duplicated *NLR* genes.

All 468 barley *NLR* genes were mapped to specific chromosomes except one. Calculation of the *NLR* gene numbers on the seven chromosomes suggested an uneven gene distribution among different chromosomes (Figure 2A). Chromosome 4 has only 16 *NLR* genes; in contrast, Chromosome 7 contains the maximal of 112 *NLR* genes, which equals to seven times of *NLR* gene number on Chromosome 4. Chromosomes 1, 2, 3, 5, and 6 each has 77, 59, 67, 66, and 69 *NLR* genes, respectively.

The distribution of *NLR* genes on barley chromosomes were further deciphered by retrieve their physical locations from the genomic gff3 file. Within each chromosome, most *NLR* genes are enriched near the telomeric region, whereas very few *NLR* genes are located at the centromere region. A total of 252 *NLR* loci were defined on the seven chromosomes, including 150 singletons and 102 multigene clusters (Figure 2B and Supplementary Table 1). The result revealed that 318 *NLR* genes are present in the 102 clusters, occupying 68% of the total *NLR* genes. This ratio is slightly lower than that in *A. thaliana* (Meyers et al., 2003). There are three *NLR* genes per cluster on average. Among the 102 defined clusters, 54 of them contains only two *NLR* genes, including 9, 8, 11, 1, 9, 8, and 8 such loci on Chromosome 1–7, respectively. The largest cluster is on chromosome 7, which has 11 *NLR* genes (Figure 2B). Over 15 clusters have more than 5 *NLR* genes.

*NLR* gene may duplicate through different mechanisms. We determined the duplication types of barley *NLR* genes using the MCScanX (Wang et al., 2012). The result shows that 74 *NLR* genes show tandem arrays, 146 are proximal duplicates (with no more than 8 interval *non-NLR* genes), 240 dispersed duplicates and eight are segmental duplicates (Figure 2C).

### Species-Specific Preservation and Amplification of Ancestral *NLR* Lineages During the Speciation of Barley and Wheat

**FIGURE 3:**
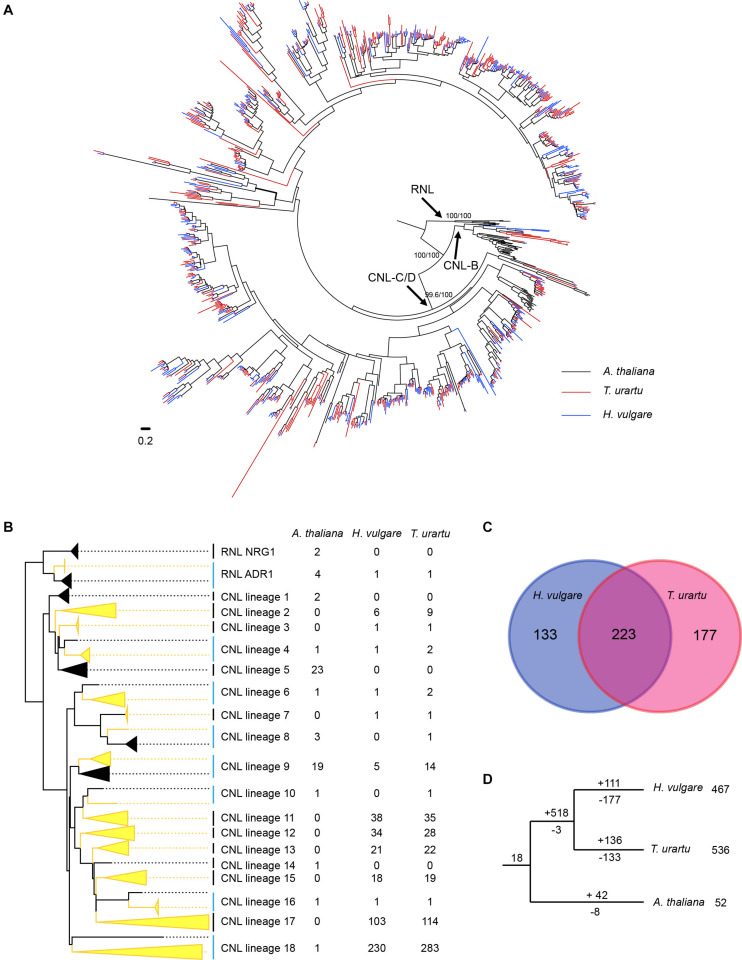
Phylogenetic and evolutionary analysis of *RNL* and *CNL* genes from barley, *T. urartu* and *A. thaliana*. **(A)** The phylogeny was constructed based on the conserved NBS domain of *CNL* and *RNL* genes from the three species. Branch support values obtained from SH-aLRT (%) and UFBoot2 (%) are labeled on basal nodes. The *CNL*-B, and *CNL*-C/D lineages are labeled according to Meyers et al. (2003). **(B)** Predicted ancestral lineages in the common ancestor of the three species. Gene number of each species on these lineages are indicated at the right of the phylogeny. **(C)** Shared and species-specific inherited of the 533 *CNL* sub-lineages that presented in the common ancestor of barley and *T. urartu*. **(D)** Duplication/loss events of the *CNL* genes during the speciation of barley, *T. urartu* and *A. thaliana*. Gene duplication/loss events are indicated by numbers with “+” or “-” on each branch, respectively.

To trace the evolutionary history of barley *NLR* genes, phylogenetic analysis was conducted by incorporating *NLR* genes from a diploid wheat *T. urartu* genome and a dicot species *A. thaliana* genome. Only *CNL* and *RNL* genes of *A. thaliana* were included in the analysis, because the two monocot species do not contain *TNL* genes. The phylogenetic analysis result revealed that *NLR* genes from the three species form two deeply separated clades with a high support value, representing the ancient divergence of *RNL* and *CNL* subclasses (Figure 3 and Supplementary Figure 1). *RNL* genes from the three species further separated into two lineages, namely *ADR1* and *NRG1*. The only *RNL* gene in barley (HORVU.MOREX.r3.5HG0438750) together with one *T. urartu RNL* gene (TRIUR3_09219) form a highly supported lineage with four *A. thaliana ADR1* genes (Supplementary Figure 1). The remaining two *A. thaliana RNL* genes form a sister lineage to *ADR1*, corresponding to the *NRG1* lineage. This topology is in accordance with the previous finding that the *RNL*-*NRG1* lineage was lost in the common ancestor of monocots (Collier et al., 2011; Shao et al., 2016; Liu et al., 2021).

*CNL* genes from the three species form two deep and well-supported clades (Figure 3A and Supplementary Figure 1). One contains the previously defined *A. thaliana CNL-B* clade genes, whereas the other one contains *A. thaliana CNL-C* and *D* clades genes (Meyers et al., 2003). Notably, only eight barley *CNL* gene and 12 *T. urartu CNL* genes are presented in the *CNL-B* clade, whereas the remaining over 400 *CNL* genes in each species are presented in the *CNL-C*/*D* clade. This phenomenon is quite different to that observed in *A. thaliana*, which has equal number of *CNL* genes in the two clades (Meyers et al., 2003). The results suggested that lineage-specific expansion of *CNL-C*/*D* genes occurred in the two monocot species.

Further reconciling the *NLR* phylogeny with species relationship revealed that at least 18 ancestral *CNL* lineages were presented in the progenitor of the three species before the divergence of monocots and eudicots (Figure 3B and Supplementary Figure 1). Among the 18 ancestral *CNL* lineages, seven (Lineage 4, 6, 8, 9, 10, 16, and 18) were inherited by *A. thaliana* and at least one of the Poaceae species. Among them, the lineages 4, 6, and 16 seem to have conservatively evolved in all three species, with no more than four genes per species (Figure 3B). In contrast, lineage 18 has expanded greatly to 230 and 283 genes in barley and *T. urartu*, respectively, whereas only maintained one copy in *A. thaliana*. The *NLR* genes in this single lineage occupies about half of all *NLR* genes in barley and *T. urartu*, providing a good example of differential expansion among different lineages. Lineage 9 experienced moderate expansion in *A. thaliana* and the two Poaceae species, with 5–19 *NLR* genes in each species.

There are three lineages only inherited by *A. thaliana* and eight lineages only inherited by barley and/or *T. urartu*, indicating that the two monocot species inherited more ancestral *CNL* lineages than *A. thaliana*. In total, the ancestor of barley and *T. urartu* inherited 15 of the 18 ancestral *CNL* lineages that emerged in the common ancestor of *A. thaliana* and the two Poaceae species. These ancestral *CNL* lineages further diverged into 533 sub-lineages before separation of barley and *T. urartu* (Figure 3C). Among them, 223 sub-lineages were maintained in both species after speciation, whereas 133 and 177 sub-lineages were only inherited by barley and *T. urartu*, respectively (Figure 3C). This means a considerable of *CNL* sub-lineages have been independently lost in the two species. Besides the gene loss events, species-specific gene duplication also occurred frequently. For example, some sub-lineages duplicated to up to ten copies in barley since it separated from *T. urartu* (Supplementary Figure 1). The species-specific gene duplication occurred more than loss of ancestral sub-lineages in *T. urartu*, which resulted in the fact that the *NLR* gene number in its current genome is larger than that in the ancestor of barley and *T. urartu*. However, in barley the *NLR* sub-lineage loss has not compensated by species-specific gene duplications, suggesting an “expansion to contraction” shift of the evolutionary pattern.

## Discussion

Plant *R* genes play vital roles in its defense against various pathogens (Xue et al., 2020). The *NLR* gene family composes the largest group of plant *R* genes (Kourelis and van der Hoorn, 2018). With the development of DNA sequencing technology, hundreds of plant genomes have been sequenced in the past 20 years, which have greatly benefitted the evolutionary analysis and functional mining of *R* genes in economically important plants (Wang et al., 2019; Liu et al., 2021). Genome-wide identification and evolutionary analysis has been performed in over 300 angiosperms since the studies in rice and *A. thaliana* genomes 20 years ago (Bai et al., 2002; Meyers et al., 2003; Liu et al., 2021). Previous studies identified less than 200 *NLR* genes from barley assemblies generated from short-read sequence sequencing strategy (Andersen et al., 2016; Habachi-Houimli et al., 2018). The number was much smaller than that in wheat, a close relative of barley that separated 11.6 million years ago (Chalupska et al., 2008). The hexaploid wheat *T. aestivum* has over 2000 *NLR* genes due to recently occurred polyploidization, whereas the diploid wheat *T. urartu* has 537 *NLR* genes. However, by improving the barley genome with long-read sequence strategy, a recently study revealed that the *NLR* gene number in barley might have been underestimated (Mascher et al., 2021).

In this study, a total of 468 *NLR* genes were identified from the improved barley genome. The abundance of *NLR* genes in barley is only slightly smaller than that in the diploid wheat *T. urartu*. Since similar methodologies were used by our and previous studies (Andersen et al., 2016; Habachi-Houimli et al., 2018), the result suggested that the great difference of *NLR* gene numbers in barley identified in the present study and previous studies should be caused by genome assembling issues of the short-read sequence strategy. This is in accordance with the result of a recent study, which showed that the updated barley genome has more *NLR* genes at the *MLA* locus than the early version genome assembly (Mascher et al., 2021). Furthermore, the wide distribution of barley *NLR* genes on the phylogeny that constructed with *NLR* genes from *T. urartu* and *A. thaliana* suggested that *NLR* gene diversity in barley is also comparable with those in *T. urartu*. Recent studies reported that some functional *NLR* genes can be transformed from wheat or barley to each other for molecular breeding (Halterman et al., 2001; Zhang et al., 2019). The high abundance and diversity of *NLR* genes in barley reported in the present study suggested that barley could be an important resource for exploring *NLR* genes to serve its relatives. Tandem duplication of *NLR* gene can generate *NLR* clusters on chromosomes, which is important for maintaining *NLR* diversity and generating novel functional *R* genes (Innes et al., 2008; Shao et al., 2014). In barley, the *MLA* locus is also a multigene cluster with several functional alleles identified (Seeholzer et al., 2010; Mascher et al., 2021). Our data revealed that 318 of identified *NLR* genes in barley form 102 clusters on its seven chromosomes, accounting for 68% of all *NLR* genes. These *NLR* clusters may serve as important reservoirs for preserving and generating of barley *NLR* diversity. Therefore, deciphering the character of chromosomal distribution and cluster arrangement of barley *NLR* genes would be helpful for map-based cloning of functional *R* genes and molecular breeding in barley.

Plant-microbe interaction is a long-term “arms race,” which can drive rapid turnover of *NLR* profiles during species-speciation (Liu et al., 2021). Therefore, *NLR* genes often exhibit rapid losses and duplications of ancestral lineages, resulting in few conserved *NLR* lineages preserved across different species. For example, only seven ancestral lineages were inherited by four legume species and maintained in a conservative manner (Shao et al., 2014). The rare long-term conservatively evolved *NLR* genes must have been constrained by conserved functions. For example, *RNL* genes function as NLR signal transducers in both *Arabidosis* and tobacco (Castel et al., 2019; Saile et al., 2020). In this study, we identified five lineages, namely lineages 4, 6, 8, 10, and 16, that conservatively evolved in both *A. thaliana* and the two Poaceae species. Interestingly, the *NLR* genes from *A. thaliana* in lineage 4 and lineage 10 are *RPS2* and *RPM1*. Proteins encoded by both genes are responsible for resistance to *Pseudomonas syringae* by monitoring the state changes of the host protein RIN4 (Bent et al., 1994; Mackey et al., 2002). Determining the close relationship of these *NLR* genes in barley to *A. thaliana* functional *R* genes and uncovering their conserved evolutionary pattern may provide clues for exploring their function in barley.

The ancestor of *T. urartu* and barley have expanded its *NLR* sub-lineage to 533 after its separation from *A. thaliana* about 100 million years ago. The majority of these *NLR* sub-lineages are descendants of *CNL* lineage 18. However, the 533 sub-lineages presented in the ancestor of *T. urartu* and barley are differently inherited by the two species. *T. urartu* only preserved 400 of these sub-lineages and duplicated to the 536 *CNL* genes in its current genome, whereas barley preserved 356 of these sub-lineages and duplicated to the 467 *CNL* genes in its current genome. Recently occurred polyploidization caused *NLR* genes in several hexaploid Triticum species expanded to more than 1000, reflecting rapidly changed *NLR* profiles after species-speciation by species-specific gene loss and duplication. Considering the transformable of functional *NLR* genes between the two species (Halterman et al., 2001; Zhang et al., 2019), the shared and species-specific *NLR* genes may further expand the cross-species pan-NLRome.

## Conclusion

Overall, a total of 468 *NLR* genes were identified from the improved barley genome, including one *RNL* subclass and 467 *CNL* subclass genes. The structure diversity, chromosomal distribution and evolutionary history of barley *NLR* genes were comprehensively analyzed. These results extended the understanding on the abundance and diversity of *NLR* genes in this important crop, which may serve as a fundamental resource for the molecular breeding of barley.

## Data Availability Statement

The original contributions presented in the study are included in the article/Supplementary Material, further inquiries can be directed to the corresponding author/s.

## Author Contributions

Z-QS conceived, designed the study, and revised the manuscript. QL and X-MJ obtained, analyzed the data, and wrote the manuscript. All authors read and approved the final manuscript.

## Conflict of Interest

The authors declare that the research was conducted in the absence of any commercial or financial relationships that could be construed as a potential conflict of interest.

## Footnotes

1 https://www.croptrust.org/crop/barley/

2 http://doi.org/10.5447/ipk/2021/3

3 http://compbio.nju.edu.cn/app/ANNA/

## Supplementary Material

Supplementary Figure 1 The full phylogeny of *NLR* genes from barley, *T. urartu* and *A. thaliana*.

Supplementary Table 1 Detailed features of *NLR* genes identified from barley genome.
