# Supplementary material for: Genome-Wide Analysis of NLR Disease Resistance Genes in an Updated Reference Genome of Barley
Source: Front Genet. 2021 May 24;12:694682. doi: 10.3389/fgene.2021.694682 (PMC8181414; doi:10.3389/fgene.2021.694682)

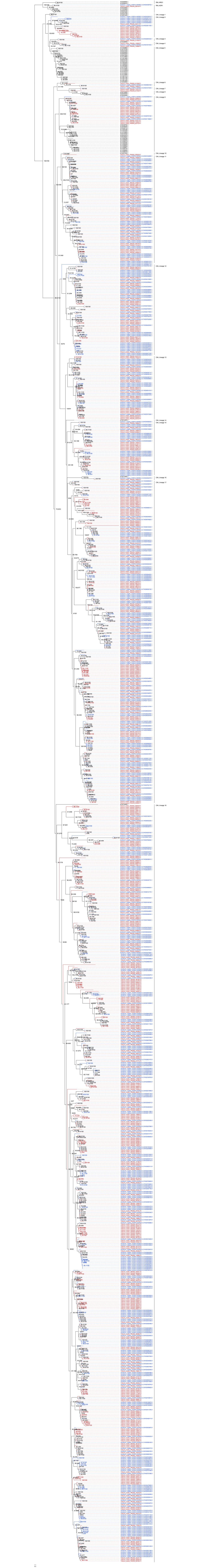

Supplement: Supplementary Figure 1 — The full phylogeny of NLR genes from barley, T. urartu and A. thaliana. [file Data_Sheet_1.PDF]
